# Within-person reproducibility of urinary bisphenol A and phthalate metabolites over a 1 to 3 year period among women in the Nurses’ Health Studies: a prospective cohort study

**DOI:** 10.1186/1476-069X-12-80

**Published:** 2013-09-13

**Authors:** Mary K Townsend, Adrian A Franke, Xingnan Li, Frank B Hu, A Heather Eliassen

**Affiliations:** 1 Channing Division of Network Medicine, Department of Medicine, Brigham and Women’s Hospital and Harvard Medical School, 181 Longwood Ave, Boston, MA 02115, USA; 2 University of Hawai’i Cancer Center, 701 Ilalo St, Honolulu, HI 96813, USA; 3 Department of Epidemiology, Harvard School of Public Health, 677 Huntington Ave, Boston, MA 02115, USA; 4 Department of Nutrition, Harvard School of Public Health, 665 Huntington Ave, Boston, MA 02115, USA

**Keywords:** Adult, Biological markers/urine, Bisphenol a, Phthalate metabolites, Reproducibility

## Abstract

**Background:**

Associations of bisphenol A and phthalates with chronic disease health outcomes are increasingly being investigated in epidemiologic studies. The majority of previous studies of within-person variability in urinary bisphenol A and phthalate metabolite concentrations have focused on reproducibility over short time periods. Long-term reproducibility data are needed to assess the potential usefulness of these biomarkers for prospective studies, particularly those examining risk of diseases with long latency periods. Low within-person reproducibility may attenuate relative risk estimates and reduce statistical power to detect associations with disease. Therefore, we assessed within-person reproducibility of bisphenol A, eight phthalate metabolites, and phthalic acid in spot urine samples over 1 to 3 years among women enrolled in two large cohort studies.

**Methods:**

Women in the Nurses’ Health Study and Nurses’ Health Study II provided two spot urine samples, 1 to 3 years apart (n = 80 women for analyses of bisphenol A; n = 40 women for analyses of phthalate metabolites; n = 34 women for analyses of phthalic acid). To measure within-person reproducibility, we calculated Spearman rank correlation coefficients and intraclass correlation coefficients for creatinine-adjusted concentrations of bisphenol A, phthalate metabolites, and phthalic acid.

**Results:**

Over 1 to 3 years, within-person variability of bisphenol A was high relative to total variability (intraclass correlation coefficient = 0.14) and rankings of bisphenol A levels between time-points were weakly correlated (Spearman correlation = 0.19). Seven of the eight phthalate metabolites and phthalic acid demonstrated moderate within-person stability over time (Spearman correlation or intraclass correlation coefficient = 0.39-0.55). Restricting analyses to first-morning urine samples did not alter results.

**Conclusions:**

Single measurements of bisphenol A in spot urine samples were highly variable within women over 1 to 3 years, indicating that investigation of associations between a single urinary bisphenol A measurement and disease risk may be challenging in epidemiologic studies. The majority of urinary phthalate metabolites and phthalic acid appeared moderately reproducible within women over time, suggesting single measurements may be useful in epidemiologic studies, although observed relative risks can be substantially attenuated.

## Background

Bisphenol A (BPA) is a man-made chemical used mainly in the manufacture of polycarbonate plastics and epoxy resins [1]. BPA-containing plastics and epoxy resins are used in many items such as containers for foods and beverages, impact-resistant safety equipment and baby bottles, toys, coatings inside metal cans, dental composites and sealants, and in color developer used in certain types of receipt paper [1,2]. In addition, BPA is present in the air, soil, and water [2]. Thus, humans may be exposed to BPA through multiple sources, including the diet, dermal contact, and inhalation [2].

Esters of phthalic acid, or phthalates, are a class of man-made chemicals used to produce plastics as well as other items. Phthalates are found in products such as floorings, clothing, packaging materials, toys, medical devices made with polyvinyl chloride (e.g., blood and enteral nutrition bags, tubing, catheters), and personal care products [1]. Since phthalates are not chemically bound to plastics, they are ubiquitous in the environment. Human exposure to phthalates occurs through ingestion, inhalation, medical procedures, and dermal absorption [1,3].

BPA and phthalates disrupt the endocrine system [2,4]. Thus, there is great scientific interest in elucidating potential pathologic effects of human exposure to these chemicals. Given the short half-lives of BPA and phthalates [5,6], epidemiologic studies examining relations between environmental exposure to these chemicals and health outcomes have focused on measurements in urine, rather than in blood where concentrations are generally low [1]. Also, since phthalates are rapidly metabolized in the body to their respective hydrolytic monoesters and oxidized monoesters [1], research studies generally measure urinary concentrations of phthalate metabolites as biomarkers of exposure to phthalates. To date, the large majority of epidemiologic studies of associations of urinary BPA and phthalate metabolites with risk of chronic conditions in adults have primarily used cross-sectional study designs. For example, studies using data from the National Health and Nutrition Examination Survey have reported modest associations of urinary BPA with cardiovascular disease [7], diabetes [8], and hypertension [9] as well as modest to strong associations of urinary phthalate metabolites with diabetes [10], endometriosis and uterine leiomyomata [11,12], and poor pulmonary function [13]. Few prospective studies have examined the relation of urinary BPA and phthalate metabolites with risk of chronic disease in adults, although one study observed a borderline-significant increased risk of incident coronary artery disease over 11 years with increasing BPA concentration measured in spot urine samples (odds ratio = 1.11 per 4.6 ng/mL increase in BPA, p = 0.06) [14].

Investigation of disease associations in prospective studies is the next step for this area of research. However, since many epidemiologic studies lack resources to collect multiple urine samples per study participant, knowledge of long-term within-person reproducibility of urinary BPA and phthalate metabolites is critical to determine whether a single measurement adequately reflects longer-term exposures. Exposures with low within-person reproducibility may attenuate relative risks and reduce statistical power to detect associations with disease [15]. Most studies examining reproducibility of urinary BPA and phthalate metabolites in adults have focused on short time periods (i.e., 2 days to 3 months) [16,17], which may not be relevant for studies examining risk of diseases with long latency periods. Four studies [18-21] that evaluated reproducibility of urinary BPA or phthalate metabolites in adults over a period longer than 3 months focused on adults aged ≤55 years attending a fertility clinic or pregnant women, among whom patterns of exposure to BPA and phthalates may be different than among older adults. Therefore, we examined within-person reproducibility of BPA, eight phthalate metabolites, and phthalic acid (PA) among women aged 33 to 78 who donated two spot urine samples 1 to 3 years apart.

## Methods

### Study design

The Nurses’ Health Study (NHS) began in 1976 when 121,700 female registered nurses, aged 30 to 55 years and living in 11 U.S. states, responded to a mailed questionnaire about their health and lifestyle [22]. In 1989, a second cohort, the NHSII, was established when 116,430 female registered nurses, aged 25 to 42 years and living in 14 U.S. states, responded to a similar questionnaire.

Details of the NHS and NHSII urine collections were published previously [23]. Briefly, in 2000 to 2001, 18,473 NHS participants were asked to collect a first morning urine sample in a polypropylene container. Women shipped the urine sample with an icepack by overnight courier to our laboratory, where it was aliquoted into polypropylene cryovials without preservative and stored in liquid nitrogen freezers. In 2002 to 2003, 2,005 of these women provided a second urine sample using the same collection and shipment methods as the initial collection.

In 1996 to 1999, 18,521 NHSII participants who had not used oral contraceptives or been pregnant or lactating within 6 months were asked to provide a first morning urine sample; 304 of these women, who were not planning to be pregnant or lactating over the next 3 years, provided an additional urine sample in 1998–2000. Collection and shipment methods were identical to those described above.

To evaluate reproducibility of BPA, phthalate metabolites, and PA, we analyzed urine samples donated 1 to 3 years (mean 2 years) apart by study participants randomly chosen from women with >1 urine collection (n = 2 samples per participant). BPA analyses included 20 NHS and 60 NHSII participants, phthalate metabolite analyses included 40 NHS participants, and PA analyses included 34 NHS participants. Participants provided implied consent by returning the questionnaires and urine samples. The Institutional Review Board of Brigham and Women’s Hospital approved this study.

### Laboratory methods

We measured BPA, eight phthalate metabolites, and PA in the spot urine samples using modifications of established methods [24,25]. The eight phthalate metabolites were: 1) monoethyl phthalate (MEP), the primary metabolite of diethyl phthalate; 2) monoisobutyl phthalate (MiBP) and 3) mono-n-butyl phthalate (MnBP), primary metabolites of dibutyl phthalates; 4) monobenzyl phthalate (MBzP), the primary metabolite of benzylbutyl phthalate; 5) mono-2-ethylhexyl phthalate (MEHP), the primary metabolite of di-2-ethylhexyl phthalate (DEHP); and three secondary metabolites of DEHP, including 6) mono-(2-ethyl-5-hydroxyhexyl) phthalate (MEHHP), 7) mono-(2-ethyl-5-oxohexyl) phthalate (MEOHP), and 8) mono-(2-ethyl-5-carboxy-pentyl) phthalate (MECPP).

We spiked 200 μL urine with 20 μL of a mixture of isotopically labeled phthalate metabolites (Table 1) and BPA-^13^C_12_, to be used as internal standards followed by treatment with β-glucuronidase and sulfatase at 37°C for 90 minutes. After acidification with 50 μL glacial acetic acid, extraction with 1.1 mL methyl tertiary butyl ether was performed. Half of the ether phase was dried with nitrogen for phthalate analysis while the second half of the ether phase was dried for BPA analysis.

**Table 1:** Parameters of phthalate metabolites and phthalic acid for orbitrap mass spectrometry quantification

**Compound name**	**Ionization mode**	**m/z**	**t**_**R **_**(minutes)**	**LOD (ng/mL)**
Monoethyl phthalate (MEP)	-ESI	193.05008	6.9	2
Mono-*n*-butyl phthalate (MnBP)	-ESI	221.08138	10.8	0.4
Monoisobutyl phthalate (MiBP)	-ESI	221.08138	10.7	0.3
Monobenzyl phthalate (MBzP)	-ESI	255.06573	11.4	0.1
Mono-2-ethylhexyl phthalate (MEHP)	-ESI	277.14398	12.8	0.5
Mono-(2-ethyl-5-hydroxyhexyl) phthalate (MEHHP)	-ESI	293.13890	10.5	0.1
Mono-(2-ethyl-5-oxohexyl) phthalate (MEOHP)	-ESI	291.12325	10.8	0.2
Mono-(2-ethyl-5-carboxy-pentyl) phthalate (MECPP)	-ESI	307.11816	10.6	0.2
Phthalic acid (PA)	-ESI	165.01858	2.63	0.1
**Internal standards**				
^13^C_4_- MEP	-ESI	197.06350	6.9	
^13^C_4_- MnBP	-ESI	225.09480	10.8	
^13^C_4_- MBzP	-ESI	259.07915	11.4	
^13^C_4_- MEHP	-ESI	281.15740	12.8	
^13^C_4_- MEOHP	-ESI	295.13666	10.8	
PA-d_4_	-ESI	169.04398	2.63	

Abbreviations: --*ESI* negative electrospray ionization, *LOD* limit of detection, *m/z* mass-to-charge ratio, *t*_*R*_ retention time.

The first half of the extract was reconstituted in 125 μL 0.1% formic acid in methanol/water (1:1) for phthalates and analyzed by orbitrap liquid chromatography-mass spectrometry (model Exactive; Thermo Electron, Waltham, Massachusetts); 0.02 mL were injected onto a BETASIL Phenyl column (150 × 2.1 mm, 3 μm, Thermo, Waltham, Massachusetts) with mobile phase A (0.1% formic acid in water) and mobile phase B (0.1% formic acid in acetonitrile) at a flow rate of 0.35 mL per minute with the following gradient: 75% A/25% B at 0 minutes, increased to 60% A/40% B at 6 minutes, then 40% A/60% B at 12 minutes and kept at the same ratio for 2 minutes, then at 14.1 minutes changed back to initial condition and equilibrium for 4 minutes. Mass detection was carried out in negative electrospray ionization mode using exact masses as detailed in Table 1. Data acquisition and analysis was performed using Xcalibur software (Thermo). Detection of the analytes was set within 10 parts per million of the calculated mass. ^13^C_4_-MBP was used as the internal standard for MECPP, MEHHP, MiBP, and MnBP since their retention times were similar; otherwise the isotope of the respective analyte was used as internal standard. Limits of detection were 0.1-1.0 ng/mL (Table 1).

BPA was analyzed by liquid chromatography-tandem mass spectrometry (model TSQ Ultra; Thermo Electron) after dansylation. The dried second fraction from above and BPA calibrators were treated with 75 μL of triethylamine (2% volume to volume in dichloromethane) and 125 μL dansyl chloride (4 mg/mL in dichloromethane). After keeping at 65°C for 30 minutes, the reaction mixture was dried under a stream of nitrogen and reconstituted in 125 μL methanol; 20 μL of the dansylated mixture was injected onto a Agilent ZORBAX SB-C18 column (3 × 50 mm, 1.8 μm; Agilent Technologies, Lexington, Massachusetts) with mobile phase A (0.1% formic acid in water) and mobile phase B (0.1% formic acid in acetonitrile) at a flow rate of 800 μL per minute with the following gradient: 40% A/60% B at 0 minutes, increased to 10% A/90% B at 10 minutes and kept at the same ratio for 1 minute, then changed back to initial condition at 11.1 minutes and equilibrium for 4 minutes. Mass detection was carried out in positive electrospray ionization mode with spray voltage at 3.5 kilovolts, capillary temperature 300°C, and sheath gas (pressure 35 units) and auxiliary gas (pressure 10 units). The divert valve was set to detector from 4–15 minutes. Signal acquisition was performed in selected reaction monitoring mode detecting the transition of mass-to-charge ratio 695 > 170 for BPA and 707 > 170 for ^13^C_12_-BPA. The limit of detection was 0.05 ng/mL.

Urinary creatinine was measured with a Roche-Cobas Mira Plus clinical chemistry analyzer (Roche Diagnostics, Indianapolis, Indiana) using a kit from Randox Laboratories (Crumlin, United Kingdom) that is based on a kinetic modification of the Jaffé reaction with a lower limit of quantitation of <15 μM/L.

To monitor assay reproducibility, we included replicate blinded quality control samples from three urine quality control pools among the participant samples to assess reproducibility of BPA (n = 16 quality control samples) and phthalate metabolites (n = 8 quality control samples), and PA (n = 6 quality control samples). Mean coefficients of variation of BPA, MEP, MiBP, MnBP, MBzP, MEHP, MEHHP, MEOHP, MECPP, and PA were 12%, 12%, 15%, 10%, 7%, 6%, 5%, 5%, 17%, and 6% at mean levels of 4, 279, 9, 31, 29, 10, 43, 31, 50, and 53 ng/mL respectively. The mean coefficient of variation for creatinine was 8% at a mean level of 0.64 mg/ml.

### Statistical analysis

To account for potential differences in urine concentrations, we divided BPA, phthalate metabolite, and PA concentrations by creatinine levels resulting in absolute excretion units (ng/mg creatinine). Additionally, we present analyte concentrations in molar units (pmol/mg creatinine). Summary phthalate metabolite measures (i.e., sum of dibutyl phthalates metabolites, sum of DEHP metabolites, and sum of all 8 metabolites) are presented in molar units only. Values were log-transformed in our analyses to account for non-normal distribution.

Within-person reproducibility of analyte levels was assessed using the Spearman rank correlation coefficient and the intraclass correlation coefficient (ICC), defined as the between-person variance divided by the sum of the within- and between-person variances. Variance components were estimated using a mixed model with participant as the random variable. An ICC ≥0.75 indicates excellent reproducibility, 0.4 to 0.75 indicates fair to good reproducibility, and <0.4 indicates poor reproducibility [26]. In additional analyses, we calculated Spearman correlations and ICCs using data from first-morning urine samples only. Also, for BPA (the study with the largest sample size), we calculated Spearman correlations and ICCs separately among women who donated urine samples <25 months apart (N = 42 women) and ≥25 months apart (N = 38 women) to explore whether reproducibility was higher in samples donated closer together in time. During laboratory analysis of PA study urine samples, one participant’s second collection measurement could not be obtained due to a technical error; we retained this participant’s first collection measurement in ICC calculations to contribute to the estimate of the between-person variance.

To assess the within-person stability of categories of analyte levels over time, we created quartile categories based on analyte distributions at each time point and cross-classification tables of analyte quartiles at time 1 versus time 2. We calculated weighted kappa statistics and 95% confidence intervals (CIs) to quantify the agreement between quartile categories defined at each time point.

## Results

Characteristics of urine samples and study participants at each time point are summarized in Table 2. Although all women were asked to collect first morning urine samples, some participants donated spot samples. Overall, 92% of NHS samples and 81% of NHSII samples were first morning urine. The large majority of study participants were non-smokers at the time of the urine collections.

**Table 2:** Characteristics of urine samples and participants in within-person reproducibility analyses

	**Bisphenol A study**		**Phthalate metabolite study**		**Phthalic acid study**	
**Variable**	**Collection 1**	**Collection 2**	**Collection 1**	**Collection 2**	**Collection 1**	**Collection 2**
Participants, N	80	80	40	40	34	33
Months between urine collections, %						
12	1		0		0	
13-18	18		5		6	
19-24	33		3		3	
25-30	23		34		41	
31-36	25		58		50	
First morning urine, n (%)	68 (85)	66 (83)	39 (98)	34 (85)	33 (97)	29 (88)
Age, years, mean (range)	48 (33–78)	50 (34–81)	66 (54–78)	69 (56–81)	66 (54–78)	69 (56–81)
White race, n (%)	76 (95)		40 (100)		34 (100)	
BMI, kg/m^2^, median (10^th^-90^th^ percentile)	25 (21–35)	25 (21–38)	24 (20–30)	23 (20–31)	24 (20–30)	23 (20–30)
Current cigarette smoker, n (%)	2 (3)	1 (1)	2 (5)	1 (3)	1 (3)	1 (3)

Abbreviation: *BMI* body mass index.

Among phthalate metabolites, MEP had the highest excretion (median 140.5, 5^th^ to 95^th^ percentile: 21.0, 894.6 ng/mg creatinine) and MiBP the lowest excretion (median 3.5, 5^th^ to 95^th^ percentile: 0.9-12.0 ng/mg creatinine) (Table 3). The excretion of BPA was the lowest of all measured analytes (median 2.7, 5^th^ to 95^th^ percentile: 0.9-9.2 ng/mg creatinine).

**Table 3:** Urinary bisphenol A, phthalate metabolite, and phthalic acid excretion distributions after adjustment for creatinine levels

**Analyte**	**Parent phthalate**	**Geometric mean**		**Median (5**^**th **^**- 95**^**th **^**percentile)**	
		**ng/mg creatinine**	**pmol/mg creatinine**	**ng/mg creatinine**	**pmol/mg creatinine**
Bisphenol A	NA	2.8	12.1	2.7 (0.9 – 9.2)	12.0 (3.8 – 40.3)
Phthalate metabolite					
MEP	DEP	135.9	700.7	140.5 (21.0 – 894.6)	724.2 (108.2 – 4611.2)
MiBP	DBP	3.6	16.1	3.5 (0.9 – 12.0)	15.6 (4.2 – 54.0)
MnBP	DBP	37.0	166.7	36.8 (10.4 – 177.0)	165.8 (46.7 – 797.4)
DBP metabolite sum^a^	DBP		190.9		177.7 (52.8 – 1157.2)
MBzP	BzBP	16.1	62.8	14.3 (4.2 – 96.9)	55.7 (16.3 – 378.4)
MEHP	DEHP	5.8	20.8	5.1 (1.5 – 26.0)	18.5 (5.3 – 93.4)
MEHHP	DEHP	35.7	121.3	31.7 (8.7 – 162.8)	107.9 (29.5 – 553.8)
MEOHP	DEHP	30.7	105.0	25.6 (7.4 – 136.2)	87.7 (25.3 – 466.5)
MECPP	DEHP	46.3	150.4	42.5 (15.8 – 214.8)	137.9 (51.4 – 697.4)
DEHP metabolite sum^a^	DEHP		421.4		361.7 (120.4 – 1520.5)
Total^b^	NA		1681.3		1566.0 (415.5 – 8687.9)
Phthalic acid	NA	88.2	531.1	90.0 (38.5 – 196.1)	542.5 (232.2 – 1181.5)

Abbreviations: *BzBP* benzylbutyl phthalate, *DBP* dibutyl phthalates, *DEHP* di-2-ethylhexyl phthalate, *DEP* diethyl phthalate, *MBzP* monobenzyl phthalate, *MECPP* mono-(2-ethyl-5-carboxy-pentyl) phthalate, *MEHHP* mono-(2-ethyl-5-hydroxyhexyl) phthalate, *MEHP* mono-2-ethylhexyl phthalate, *MEOHP* mono-(2-ethyl-5-oxohexyl) phthalate, *MEP* monoethyl phthalate, *MiBP* monoisobutyl phthalate, *MnBP* mono-n-butyl phthalate, *NA* not applicable.

^a^DBP metabolite sum = MiBP + MnBP; DEHP metabolite sum = MEHP + MEHHP + MEOHP + MECPP.

^b^Sum of 8 phthalate metabolites.

In all samples, as well as first-morning urine samples, we observed high within-person variability relative to total variability of BPA (ICC = 0.14 and 0.15, respectively) (Table 4). In addition, rankings of BPA excretion 1 to 3 years apart were weakly correlated (Spearman correlation = 0.19 for all samples and 0.23 for first-morning urine samples). In additional analyses, we separately evaluated reproducibility of BPA among women who donated urine samples <25 versus ≥25 months apart. Within-person variability relative to total variability remained high among both sub-groups of women, but appeared slightly lower among women who donated samples closer (ICC = 0.23, 95% CI 0.06, 0.60) than further apart in time (ICC = 0.06, 95% CI 0.00, 0.94), although CIs for the ICCs were wide. Spearman correlations were similar in these sub-groups (0.16 among samples donated <25 months apart and 0.23 among samples donated ≥25 months apart).

**Table 4:** Within-person reproducibility of urinary bisphenol A, phthalate metabolites, and phthalic acid over 1–3 years

	**All samples**				**First morning urine samples only**			
**Analyte**^**a**^	**Spearman correlation**	**Between-person variation**	**Within-person variation**	**Intraclass correlation (95% CI)**	**Spearman correlation**	**Between-person variation**	**Within-person variation**	**Intraclass correlation (95% CI)**
Bisphenol A	0.19	0.08	0.50	0.14 (0.03, 0.49)	0.23	0.09	0.53	0.15 (0.03, 0.54)
Phthalate metabolites								
MEP	0.41	0.40	0.80	0.33 (0.13, 0.63)	0.53	0.48	0.61	0.44 (0.21, 0.70)
MiBP	0.47	0.31	0.72	0.30 (0.10, 0.62)	0.47	0.31	0.77	0.29 (0.08, 0.64)
MnBP	0.55	0.34	0.31	0.53 (0.31, 0.73)	0.44	0.29	0.35	0.45 (0.22, 0.71)
DBP sum^b^	0.51	0.31	0.37	0.45 (0.23, 0.69)	0.40	0.26	0.43	0.38 (0.15, 0.67)
MBzP	0.43	0.32	0.64	0.33 (0.12, 0.63)	0.46	0.32	0.71	0.31 (0.11, 0.64)
MEHP	0.03	0.15	0.93	0.14 (0.01, 0.67)	0.08	0.18	0.94	0.16 (0.02, 0.68)
MEHHP	0.18	0.43	0.58	0.43 (0.21, 0.68)	0.21	0.48	0.58	0.45 (0.22, 0.70)
MEOHP	0.30	0.44	0.62	0.42 (0.20, 0.67)	0.41	0.50	0.59	0.46 (0.24, 0.70)
MECPP	0.20	0.29	0.46	0.39 (0.17, 0.66)	0.27	0.36	0.41	0.47 (0.24, 0.71)
DEHP sum^b^	0.20	0.31	0.51	0.38 (0.16, 0.65)	0.30	0.35	0.49	0.42 (0.19, 0.68)
Total^c^	0.39	0.27	0.41	0.40 (0.18, 0.66)	0.54	0.32	0.31	0.51 (0.28, 0.73)
Phthalic acid	0.41	0.11	0.15	0.41 (0.18, 0.69)	0.40	0.10	0.15	0.41 (0.16, 0.71)

Abbreviations: *CI* confidence interval, *DBP* dibutyl phthalates, *DEHP* di-2-ethylhexyl phthalate, *ICC* intraclass correlation coefficient, *MBzP* monobenzyl phthalate, *MECPP* mono-(2-ethyl-5-carboxy-pentyl) phthalate, *MEHHP* mono-(2-ethyl-5-hydroxyhexyl) phthalate, *MEHP* mono-2-ethylhexyl phthalate, *MEOHP* mono-(2-ethyl-5-oxohexyl) phthalate, *MEP* monoethyl phthalate, *MiBP* monoisobutyl phthalate, *MnBP* mono-n-butyl phthalate.

^a^All values were adjusted for creatinine (pmol/mg creatinine) and natural log-transformed. Mean (range) time between collections was 2 (1–3) years.

^b^DBP sum = MiBP + MnBP; DEHP sum = MEHP + MEHHP + MEOHP + MECPP.

^c^Sum of 8 phthalate metabolites.

Overall, most phthalate metabolites (MEP, MiBP, MnBP, MBzP, MEHHP, MEOHP, and MECPP) and PA demonstrated fair or nearly fair within-person stability over time (i.e., Spearman correlation or ICC = 0.39-0.55) (Table 4). There was no correlation between rankings of MEHP measurements 1 to 3 years apart and high within-person variability in MEHP relative to total variability (Spearman correlation = 0.03, ICC = 0.14). Spearman correlations and ICCs generally were similar or slightly higher when restricting to first-morning urine samples.

We also evaluated the agreement between quartile categories defined by the analyte distribution at each time point. Levels of BPA and MEHP, which had the lowest ICCs, were classified in the same quartile at both time points for 28% of women. The percentage of women with analyte levels in the lowest quartile at time 1 whose levels were also in the lowest quartile at time 2 was 45% for BPA and 30% for MEHP. Among women with analyte levels in the highest quartile at time 1, the percentage of women with analyte levels in the highest quartile at time 2 was 25% for BPA and 20% for MEHP. The weighted kappa statistic was 0.08 for both BPA and MEHP (95% CIs = -0.08, 0.24 for BPA and -0.14, 0.30 for MEHP) (Table 5). For MnBP, which had the highest ICC, 45% of women were classified in the same quartile at time 1 and time 2. Sixty percent of women with MnBP levels in the lowest quartile at time 1, also had MnBP levels in the lowest quartile at time 2. Among women with MnBP levels in the highest quartile at time 1, 50% had levels in the highest quartile at time 2 (weighted kappa = 0.40, 95% CI: 0.19, 0.61).

**Table 5:** Weighted Kappa statistics summarizing agreement between analyte quartile categories determined at each time point

**Analyte**	**Weighted Kappa statistic (95% CI)**
Bisphenol A	0.08 (−0.08, 0.24)
Phthalate metabolite	
MEP	0.28 (0.05, 0.51)
MiBP	0.24 (0.04, 0.44)
MnBP	0.40 (0.19, 0.61)
DBP metabolite sum^a^	0.36 (0.14, 0.58)
MBzP	0.20 (−0.01, 0.41)
MEHP	0.08 (−0.14, 0.30)
MEHHP	0.08 (−0.16, 0.32)
MEOHP	0.20 (−0.03, 0.43)
MECPP	0.12 (−0.11, 0.35)
DEHP metabolite sum^a^	0.12 (−0.10, 0.34)
Total^b^	0.20 (−0.03, 0.43)
Phthalic acid	0.40 (0.14, 0.66)

Abbreviations: *BzBP* benzylbutyl phthalate, *CI* confidence interval, *DBP* dibutyl phthalates, *DEHP* di-2-ethylhexyl phthalate, *DEP* diethyl phthalate, *MBzP* monobenzyl phthalate, *MECPP* mono-(2-ethyl-5-carboxy-pentyl) phthalate, *MEHHP* mono-(2-ethyl-5-hydroxyhexyl) phthalate, *MEHP* mono-2-ethylhexyl phthalate, *MEOHP* mono-(2-ethyl-5-oxohexyl) phthalate, *MEP* monoethyl phthalate, *MiBP* monoisobutyl phthalate, *MnBP* mono-n-butyl phthalate.

^a^DBP metabolite sum = MiBP + MnBP; DEHP metabolite sum = MEHP + MEHHP + MEOHP + MECPP.

^b^Sum of 8 phthalate metabolites.

## Discussion

We observed poor within-person stability of urinary BPA over 1 to 3 years among NHS and NHSII participants, the majority of whom donated first-morning urine samples. Seven phthalate metabolites (MEP, MiBP, MnBP, MBzP, MEHHP, MEOHP, MECPP) and PA demonstrated fair or nearly fair within-person reproducibility over time. Between-person variation was greater than within-person variation (i.e., ICC >0.5) for MnBP only.

Most, but not all [18-21], previous studies of within-person reproducibility of urinary BPA and phthalate metabolites examined stability over a period of 3 months or less and in adults aged less than 60 years [16]. Regarding urinary BPA, high intra-individual variation relative to total variation has been found over 1 week among 8 adults aged 26–58 years (ICC = 0.23 in first-morning urine samples) [27] as well as over approximately 6 months among 389 pregnant women (ICC = 0.1 in spot urine samples) [19]. However, one study of 60 premenopausal women aged 21–42 years found moderate reproducibility (ICC = 0.43) of BPA measured in first-morning urine samples over 4 weeks [28]. Similar to our results, Braun et al. [20] calculated an ICC of 0.23 for repeated BPA measurements in pre-pregnancy spot urine samples donated <1 week to over 2 years apart (median 12 weeks apart) from 137 women aged 18–45 years. Regarding urinary phthalate metabolites, our ICC estimates for reproducibility of MEP, DBP metabolites, MBzP, and MEHP over 1 to 3 years generally were lower than those reported in studies examining reproducibility of these metabolites in men and non-pregnant women over periods of 3 months or less [29-31]. However, our results for DBP metabolites, MBzP, and MEHP were similar to those of Braun et al. [20], who analyzed reproducibility in samples donated up to 110 weeks apart. For example, Braun et al. reported ICCs ranging from 0.35-0.40 for DBP metabolites and MBzP, and an ICC of 0.11 for MEHP [20]. Interestingly, within-person reproducibility over time of MEHHP, MEOHP, and MECPP tended to be higher in our study than in shorter duration studies, which found ICCs ranging from 0.13-0.25 [21,30], or the longer-duration study by Braun et al., which reported an ICC of 0.11 for the sum of DEHP metabolites [20].

The half-lives of BPA and phthalate metabolites are relatively short (<6 to 15 hours) [5,6]. Also, most women in our study donated first-morning urine samples, which likely reflect exposure the previous evening. Thus, habitual exposure to sources of BPA and phthalates at about the same time each day would likely be necessary to observe reasonable within-person reproducibility in our study. The moderate correlations we observed for diethyl phthalate and dibutyl phthalates metabolites may reflect habitual use of personal care products (primary sources of diethyl phthalate and dibutyl phthalates [32]). Likewise, diet generally follows a long-term consistent pattern [33], possibly explaining moderate correlations we observed for the primary metabolite of benzylbutyl phthalate and secondary metabolites of DEHP, which may contaminate foods via the soil [34] as well as during processing and storage [3]. The primary metabolite of DEHP, MEHP, has the shortest half-life of the DEHP metabolites (5 hours [5]) and is mostly converted to the secondary metabolites via oxidative metabolism [1], which may explain its poor long-term reproducibility. PA, a non-specific phthalate metabolite, was moderately reproducible within women over time, reflecting the moderate correlations observed for the majority of phthalate metabolites.

Food packaged in polycarbonate or epoxy-lined containers is a primary source of BPA exposure [1]. Yet, unlike metabolites of benzylbutyl phthalate and DEHP, which are also found mainly in foods, we observed poor within-person stability of BPA over 1 to 3 years. The reason for this discrepancy is unclear, however, there are many potential sources of human exposure to BPA [2] and variability in non-food sources (e.g., handling of thermal receipts) may contribute to poor within-person reproducibility over time.

Several limitations of our study should be considered. First, variation in analyte levels due to delayed processing of urine samples and laboratory variability is incorporated into the ICCs. However, previous studies demonstrated excellent stability of BPA and phthalate metabolites in urine samples stored at room temperature up to 2 days, and coefficients of variation for most analytes were ≤15%, suggesting that ICCs were not substantially affected by these sources of error [35,36]. In addition, the number of women included in our studies, particularly the phthalate metabolite and PA studies, was relatively small (N = 33-80), which contributed to wide CIs for the ICC estimates; thus it is important that these results are interpreted cautiously. Finally, our estimates of long-term reproducibility of BPA and phthalate metabolites in spot urine samples are specific to NHS and NHSII participants and may not be generalizable to other populations with different exposure patterns.

## Conclusions

Overall, our observation of low reproducibility of urinary BPA excretion over 1 to 3 years indicates evaluation of associations with disease risk in epidemiologic studies may be challenging when only a single spot urine measurement is available. Indeed, according to the Spearman-Brown prophecy formula [37], our ICC estimate for BPA suggests approximately 5 spot measurements would be required to achieve a fair ICC of 0.4. Single measurements of certain phthalate metabolites and PA may be acceptable for prospective epidemiologic studies of diseases with long latency periods. Since within-person correlations were moderate, biomarker-disease associations will likely be attenuated and measurement error correction techniques may be necessary to understand the full magnitude of the impact of phthalates on health.

## Abbreviations

BPA: Bisphenol A; BzBP: Benzylbutyl phthalate; C: Carbon; CI: Confidence interval; CV: Coefficient of variation; DBP: Dibutyl phthalates; DEHP: Di-2-ethylhexyl phthalate; DEP: Diethyl phthalate; ICC: Intraclass correlation coefficient; LCMS: Liquid chromatography mass spectrometry; MBzP: Monobenzyl phthalate; MECPP: Mono-(2-ethyl-5-carboxy-pentyl) phthalate; MEHHP: Mono-(2-ethyl-5-hydroxyhexyl) phthalate; MEHP: Mono-2-ethylhexyl phthalate; MEOHP: Mono-(2-ethyl-5-oxohexyl) phthalate; MEP: Monoethyl phthalate; MiBP: Monoisobutyl phthalate; NHS: Nurses’ Health Study.

## Competing interests

The authors declare that they have no competing interests.

## Authors’ contributions

MKT participated in the design and coordination of the study, performed the statistical analysis, and drafted the manuscript. AAF designed the methods to measure BPA, phthalate metabolites, and PA, and revised the manuscript critically for important intellectual content. XL implemented the methods to measure BPA, phthalate metabolites, and PA, and revised the manuscript critically for important intellectual content. FBH participated in the design of the study and revised the manuscript critically for important intellectual content. AHE conceived of the study, and participated in its design and coordination, and helped draft the manuscript. All authors read and approved the final manuscript.
